# Structural Features Related to Affective Instability Correctly Classify Patients With Borderline Personality Disorder. A Supervised Machine Learning Approach

**DOI:** 10.3389/fpsyt.2022.804440

**Published:** 2022-02-28

**Authors:** Alessandro Grecucci, Gaia Lapomarda, Irene Messina, Bianca Monachesi, Sara Sorella, Roma Siugzdaite

**Affiliations:** ^1^Clinical and Affective Neuroscience Lab, Department of Psychology and Cognitive Sciences (DiPSCo), University of Trento, Rovereto, Italy; ^2^Center for Medical Sciences - CISMed, University of Trento, Trento, Italy; ^3^Department of Psychology, Science Division, New York University of Abu Dhabi, Abu Dhabi, United Arab Emirates; ^4^Universitas Mercatorum, Rome, Italy; ^5^MRC Cognition and Brain Sciences Unit, University of Cambridge, Cambridge, United Kingdom

**Keywords:** Borderline Personality Disorder, bipolar disorder, machine learning, multi-voxel pattern analysis, affective instability

## Abstract

Previous morphometric studies of Borderline Personality Disorder (BPD) reported inconsistent alterations in cortical and subcortical areas. However, these studies have investigated the brain at the voxel level using mass univariate methods or region of interest approaches, which are subject to several artifacts and do not enable detection of more complex patterns of structural alterations that may separate BPD from other clinical populations and healthy controls (HC). Multiple Kernel Learning (MKL) is a whole-brain multivariate supervised machine learning method able to classify individuals and predict an objective diagnosis based on structural features. As such, this method can help identifying objective biomarkers related to BPD pathophysiology and predict new cases. To this aim, we applied MKL to structural images of patients with BPD and matched HCs. Moreover, to ensure that results are specific for BPD and not for general psychological disorders, we also applied MKL to BPD against a group of patients with bipolar disorder, for their similarities in affective instability. Results showed that a circuit, including basal ganglia, amygdala, and portions of the temporal lobes and of the orbitofrontal cortex, correctly classified BPD against HC (80%). Notably, this circuit positively correlates with the affective sector of the Zanarini questionnaire, thus indicating an involvement of this circuit with affective disturbances. Moreover, by contrasting BPD with BD, the spurious regions were excluded, and a specific circuit for BPD was outlined. These results support that BPD is characterized by anomalies in a cortico-subcortical circuit related to affective instability and that this circuit discriminates BPD from controls and from other clinical populations.

## Introduction

The diagnosis of personality disorders based only on observable signs and symptoms is highly problematic considering the difficulties in distinguishing trait-dependent manifestations from active symptoms common to other mental disorders (1). One case in point is Borderline Personality Disorder (BPD), which is not only the most commonly diagnosed personality disorder affecting presumably 2% of the population (2) but also one of the most problematic diagnostic categories (1). Relevant dimensions that characterize BPD are affective difficulties, socio-interpersonal difficulties, and impulsivity (3, 4), which may lead to extremely diversified pattern of symptoms across patients (5). Moreover, the high comorbidity—e.g., with eating, abuse, personality, and affective disorders (2, 6)—and the insidious overlap of symptoms with other psychopathology (1) contribute to the difficulty of BPD diagnosis, with high rate of misdiagnosis for this disorder (7).

The problem of reliable diagnosis in psychiatry has been previously addressed in the literature (8), and affective neuroscience has been identified as a field that can crucially contribute to the overcoming of current limitations in available diagnostic systems through the detection of neurobiological markers for specific psychopathological conditions. Neuroimaging investigations of BPD have provided important insights concerning its neural correlates but limited contributions for the discrimination of healthy and pathological conditions. Structural alterations have been found in the prefrontal cortex and in several limbic structures (9). Related to these structural alterations, functional alterations consistent with the clinical manifestations of the disorder have been identified: Altered activity of dorsolateral prefrontal and limbic brain regions in response to emotional stimuli is consistent with the conceptualization of BPD as an emotion dysregulation disorder (9–13); the hyperactivity of the default mode network areas at rest is consistent with difficulties in both interpersonal and affective regulation (13–16). Structural studies partially confirm alterations in some of these regions (9, 17–22). Structural alterations have been previously reported in patients with BPD for what concerns the thalamus (6, 21, 23), the amygdala (9, 24–26), and the fusiform area (27). However, previous studies suffer from major limitations. First, they have used mass univariate analysis that examines each voxel in isolation and does not take into account statistical dependencies among voxels (28–30). Moreover, individual differences were not taken into consideration, as the average of individuals inside each group was only considered. In some cases, region of interest (ROI) analyses were used instead of whole-brain approaches, thus limiting results on a limited set of *a priori*–defined regions (31). Last but not least, these results could not be tested for generalization to new unobserved cases.

An alternative approach that has proven extremely useful for diagnostic classification of subjects on the basis of MRI signal patterns is the use of machine learning for the separation of patients from healthy controls (HCs). Machine learning, also called multi-voxel pattern analysis (MVPA) in the context of neuroscience, can dramatically increase the sensitivity of human brain imaging by accumulating information across multiple voxels of MRI signal, i.e., by taking into account the information contained in a distributed spatial pattern of brain activity rather than a single voxel or location (32). A commonly applied implementation of machine learning is the use of a classification algorithm that is trained to distinguish between two classes of data using whole-brain pattern-based information. Such techniques have proven extremely useful for the decoding of between-subject classification of brain imaging data in a number of psychiatric and neurological diseases, reaching a good classification (from 60 to 90%) in the cases of depression (33), schizophrenia (34), and social anxiety disorder (35). Among classification algorithms, the Multiple Kernel Learning (MKL) algorithm has the additional advantage to allow the identification of the most relevant sources for the classification, e.g., brain regions contributing to the model. Authors have recently used MKL to make predictions based on anatomical localization (36, 37) and to help to determine which are the most relevant brain regions that contribute to group classification to predict differential diagnosis between mood disorders (38).

The first aim of the present study is to explore, for the first time, the potentiality of MKL for the diagnostic classification of patients with BPD on the basis of their brain structural features. In line with this aim, we applied MKL to the classification of patients with BPD and HCs. We hypothesized that some of the brain structures previously identified in separate studies can effectively discriminate these groups of participants. Because MKL allows the understanding of which brain structures, among all areas, maximally discriminate the two groups in the classifier, we were also interested in understanding which brain structures are more relevant for the understanding of neurobiological features of BPD. We hypothesized to find a widely distributed circuit including portions of the orbitofrontal cortex (OFC) and of the temporal lobe, for their relations with affective disturbances and lack of control over emotions, as well as subcortical structures such as the amygdala and the basal ganglia for emotion dysregulation and impulsivity displayed by patients with BPD.

A second relevant aspect of BPD diagnosis concerns its specificity compared with other forms of psychopathologies. One of the most critical aspects of differential diagnosis regards the consideration of affective symptomatology as a manifestation of a clinical syndrome, instead of a more general personality impairment. Typically, patients with BPD seems be at increased risk of being misdiagnosed with bipolar disorder (BD) (7). Affective disturbances have been described as a core feature of both disorders (39, 40). Mood swings and anger reactions typically described in BPD can be easily observed also in maniac episodes of the BD (41). Similarly, the impulsiveness observed in maniac episodes is frequently considered as a core aspect of borderline personality (29, 30, 42–44). In a meta-analysis (45), abnormalities in the amygdala and parahippocampal gyrus were reported, and a smaller volume of the right medial OFC was detected in both BD and BPD. In another study, differences consisting in more volumetric alterations and larger diffusion in BD (involving several cortical and subcortical structures) than BPD (confined to mainly fronto-limbic regions) have been found. In a recent study, Lapomarda et al. (29) applied unsupervised machine learning to compare BPD, BD, and controls. A blind source separation method known as Independent Component Analysis (ICA) (28–31, 46–48) was applied to gray and white matter. Compared with controls, patients with BD increased gray matter in a network involving mostly subcortical structures and cerebellar areas, possibly related to abnormal mood. In contrast, patients with BPD showed milder alterations compared with patients with BD and controls. Moreover, BPD differed from BD and controls for a white matter circuit including frontal-parietal and temporal regions possibly associated with dysfunctional top-down emotion regulation (29, 49–51). However, one limitation of the method used in that study is that the unsupervised machine learning used (ICA) is not suitable for classification or for creating biomarkers to predict the diagnosis of new cases as it strictly depends on the sample used. Moreover, ICA was applied simultaneously to BPD, BD, and HC. Thus, the results found for BPD depended on the simultaneous comparison of all three groups. Thus, following the first aim, we intended to apply the same methodology but this time to classify BPD against BD. This will let us understand which brain structures maximally discriminate the two groups and may help the differential diagnoses of such patients. We predicted that the classifier can correctly classify BPD from BD and that similar regions involved in the classification of BPD against HC will discriminate BPD against BD. Moreover, by subtracting the circuit that correctly separates BPD from BD, from the circuit that separates BPD from HC, a possible neural substrate to serve as a starting point for developing a biomarker specific for BPD can be outlined. This circuit may serve as a baseline for future investigations intended to develop a biomarker for correctly diagnosing BPD from structural brain features.

## Methods

### Participants

We selected 20 patients with BPD (*M*_age_ = 35.75, *SD*_age_ = 8.61), 30 patients with BD type I (BD; *M*_age_ = 37.17, *SD*_age_ = 8.64), and 45 healthy participants as controls (HC; *M*_age_ = 36.80, *SD*_age_ = 8.43), matched for age and sex. All the data were extracted from the shared OpenNeuro database (52). Demographic information about participants is displayed in Table 1.

**Table 1:** Demographic information about participants.

	**BPD**	**BD**	**HC**	***p*-values**
Participants	20	30	45	
Age (years)	*M*_age_ = 35.75 (±8.61)	*M*_age_ = 37.17 (±8.64)	*M*_age_ = 36.80 (±8.43)	*F*_(2,92)_ = 0.172 *p* = 0.842
Gender	*F* = 17	*F* = 21	*F* = 34	*F*_(2,92)_ = 0.725 *p* = 0.487
Education	≥8	≥8	≥8	
Screening	Neurological disease, psychoactive substance, mental illness (SCID-II, SCID-IV)	Neurological disease, psychoactive substance, mental illness (SCID-II, SCID-IV)	Neurological disease, psychoactive substance, mental illness (SCID-II, SCID-IV)	
Exclusion criteria	Diagnosis in at least two different categories, pregnancy, MRI contraindications, neurological disease	Diagnosis in at least two different categories, pregnancy, MRI contraindications, neurological disease	Diagnosis for any psychiatric or neurologic disease, pregnancy, MRI contraindications	

*The presented values for “Age” and “Education” are the relative arithmetic averages of years. Values in round brackets are the standard deviations*.

Patients with BPD were selected from Clinical Research Imaging Centre in Edinburgh (OpenNeuro database, accession number ds000214). The recruitment took place in outpatient and support services from around Edinburgh. The diagnosis was verified using Structured Clinical Interview for DSM-IV (SCID-II). The Zanarini Rating Scale for BPD (ZAN-BPD) was administered to assess the current symptoms. Exclusion criteria included pregnancy, MRI contraindications, diagnosis of a psychotic disorder, and current illicit substance dependence. All participants gave written informed consent approved by the Lothian National Health Service Research Ethics Committee. Patients with BPD were acquired with a 3T Siemens Magneton Verio with TR = 2,300 (ms), TE = 2.98 (ms), and 160 slices. Patients with BD and HCs were selected from UCLA Consortium for Neuropsychiatric Phenomics (OpenNeuro database, accession number ds000030). They were recruited *via* community advertisements in the Los Angeles area. Self-reported history of psychopathology was verified with the SCID-IV (53). Inclusion criteria comprised the following: at least 8 years of education, no history of head injury with loss of consciousness or cognitive sequelae, no use of psychoactive medications or substance dependence within past 6 months, and no history of major mental illness. Participants were excluded if they had history of significant medical illness, contraindications for MRI, and mood-altering medication on scan day (based on self-report). All participants gave written informed consent approved by the University of California, Los Angeles Institutional Review Board. A high-resolution T1-weighted 3D magnetization prepared rapid gradient echo scan was acquired for each participant. HCs and patients with BD were acquired with a 3T Siemens Magneton Trio with TR = 1,900 (ms), TE = 2.26 (ms), and 176 slices.

### Preprocessing

After quality check of the images to exclude artifacts and before any analyses, all data were preprocessed with the same pipeline using the segmentation routines provided by the Computational Anatomy Toolbox (CAT12, http://www.neuro.uni-jena.de/cat/), a toolbox available for SPM12 software (http://www.fil.ion.ucl.ac.uk/spm/software) in the MATLAB environment. Segmentation of gray and white matter and cerebrospinal fluid was thus obtained. Modulated normalized writing option was chosen. Diffeomorphic Anatomical Registration through Exponential Lie algebra (DARTEL) tool, a potential alternative to SPM's traditional registration approaches that operates using a whole-brain approach, was used (47, 48, 54). Normalization to MNI space with spatial smoothing (full width at half maximum of Gaussian smoothing kernel [8, 8, 8]) was then applied on DARTEL images.

### Data Analysis

MVPA on the basis of MKL method was carried out in the Pattern Recognition for Neuroimaging Toolbox (PRoNTo) (55, 56). Between-group analyses, with patients with BPD against HC, and then patients with BPD against patients with BD, were entered as two classes in separate MKL analyses with the preprocessed gray matter images. In MKL, heterogenous kernels are linearly combined (57), to define a decision boundary. MKL has been shown to enhance the interpretability of the decision function and improve performances (57). Whole-brain analyses were performed using a general mask provided inside PRoNTo. Data were mean centered and normalized, and age and gender were regressed out. The predictive function was defined during a training phase where the algorithm learned patterns from the provided data to predict a label (diagnosis). Whereas, during a test phase, the algorithm is used to predict outcome in an independent dataset. Leave-one-subject-out cross-validation was performed, making the test set independent from the training set. Each class accuracy was calculated averaging classification results across all the folds of cross-validation (55). Statistical significance of the classifications was tested using permutation testing with 1,000 permutations with random assignment of group class to input image. The resulting null-hypothesis distribution was used to calculate the *p*-value of the accuracies or the proportion of permutations that yielded a greater accuracy than the accuracy found for the classification models. The Automated Anatomical Labeling [AAL; (58)] atlas, built using the WFU- PickUp Atlas toolbox of SPM and consisting of 116 brain regions, was used to explore regional contribution of each classification model. In MKL approach, being a hierarchical model of the brain, it was possible to derive weight contribution of each region to the decision function. Regions were ranked according to their contribution to the model and averaged across folds. Only regions with >1% contribution to the decision function f are displayed. Through the pair-wise classification, common, and unique structural features among the two contrasts were identified [see (59), for a similar approach], using the formula BPD specificity = (BPD ≠ HC) ∧ (BPD ≠ BD). If one structural area separated BPD against HC, and the same also separated BPD against BD, this feature was selected as a specific neural abnormality uniquely associated with BPD. By contrast, regions that separated BPD against HC, but not BPD against BD, were excluded (in other words, this region may be similarly affected in both BPD and BD, and so, it cannot be considered specific for BPD). Surf Ice software was used to plot the brain maps (https://www.nitrc.org/projects/surfice/).

## Results

No significant differences were found for age [*F*_(2,92)_ = 0.172, *p* = 0.842], gender [*F*_(2,92)_ = 0.725, *p* = 0.487], HDRS, and medication load comparing BPD, HC, and BD (all *p* > 0.05).

### BPD Against Controls

**Figure 1:**
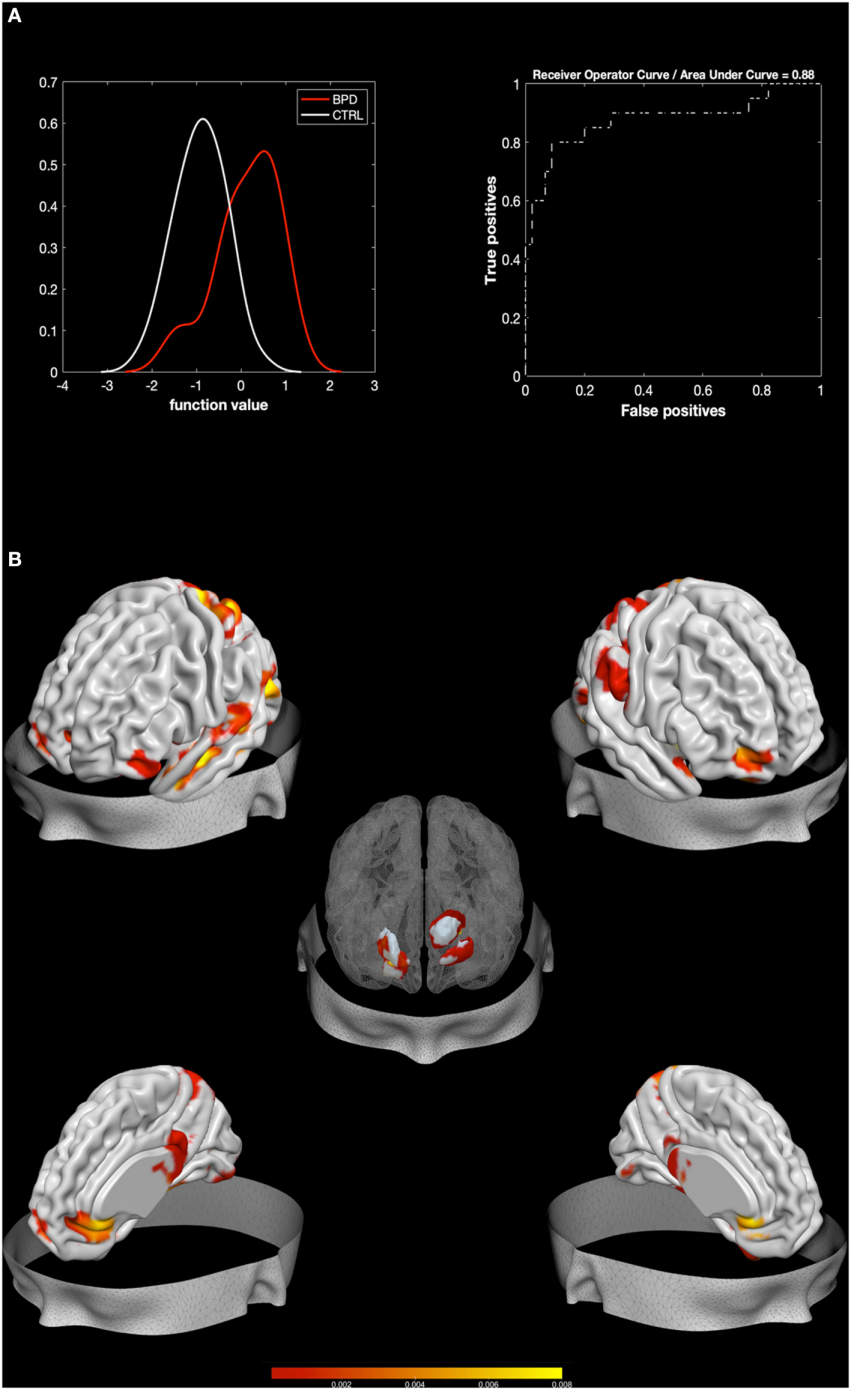
Results from BPD against HC. Multiple Kernel Learning machine classification of patients with Borderline Personality Disorder (BPD) and healthy controls (HC) based on structural (GM) features. **(A)** Left: Density version of histogram plot of function values. Right: Receiver Operator Curve, Areas Under the Curve = 0.88. ROI weights in percentage and in voxel size are displayed in the two bar plots. **(B)** Surface plots, including subcortical reconstruction of the significant regions.

The MKL returned a total accuracy of 84.62%, bipolar disorder (BA) of 76.39% (*p* = 0.002), class predictive values of 91.6–97.78%, and AUC value of 88% (Figure 1). Model performance significantly exceeds the threshold of randomly guessing the labels, thus confirming that the algorithm has successfully learned a predictive function (56). A positive correlation was found between Zanarini affective sector scores and betas extracted from the classifier (*r* = 0.45, *p* = 0.047), thus confirming a relation between the circuit that predicts BPD and affective disturbances.

Regions with larger contribution to the model (weight > 1%) were in order of importance: the right Putamen, the left thalamus, the right fusiform gyrus, the right amygdala, the lingual gyrus, the right middle and superior OFC, the left pallidum, the left fusiform gyrus, and portions of the cerebellum (see Figure 1; Table 2). To characterize the direction of these areas, simple voxel-to-voxel comparisons were computed. Raw data were extracted from the gray matter (GM) images of both BPD and HC after masking for the circuit found by MKL analysis. A threshold of 0.001 uncorrected was used. This analysis showed that all areas were characterized by greater GM for BPD relative to HC, except the putamen, the pallidus, and the thalamus, which showed the opposite trend.

**Table 2:** Main regions derived from the classification of BPD against controls.

**ROI labels**	**ROI weight (%)**	**ROI size (voxels)**
Putamen_R	21.1314	2,560
Thalamus_L	11.0615	2,420
Temporal_Mid_L	10.4139	11,409
Fusiform_R	8.3912	5,731
Amygdala_R	6.7290	571
Lingual_R	6.6867	5,574
Frontal_Sup_Orb_R	5.9255	1,352
Pallidum_L	5.5983	637
Frontal_Mid_Orb_R	4.2866	1,583
Occipital_Mid_R	3.9392	4,649
Parietal_Sup_R	3.3007	3,557
Vermis_7	1.9245	458
Fusiform_L	1.7186	5,282
Cerebelum_Crus2_L	1.6214	4,105
Cerebelum_7b_L	1.5011	863
Heschl_L	1.2470	549

*ROI labels are derived from the AAL atlas. Please note that only regions whose contribution exceeded the 1% are displayed*.

### BPD Against BD

**Figure 2:**
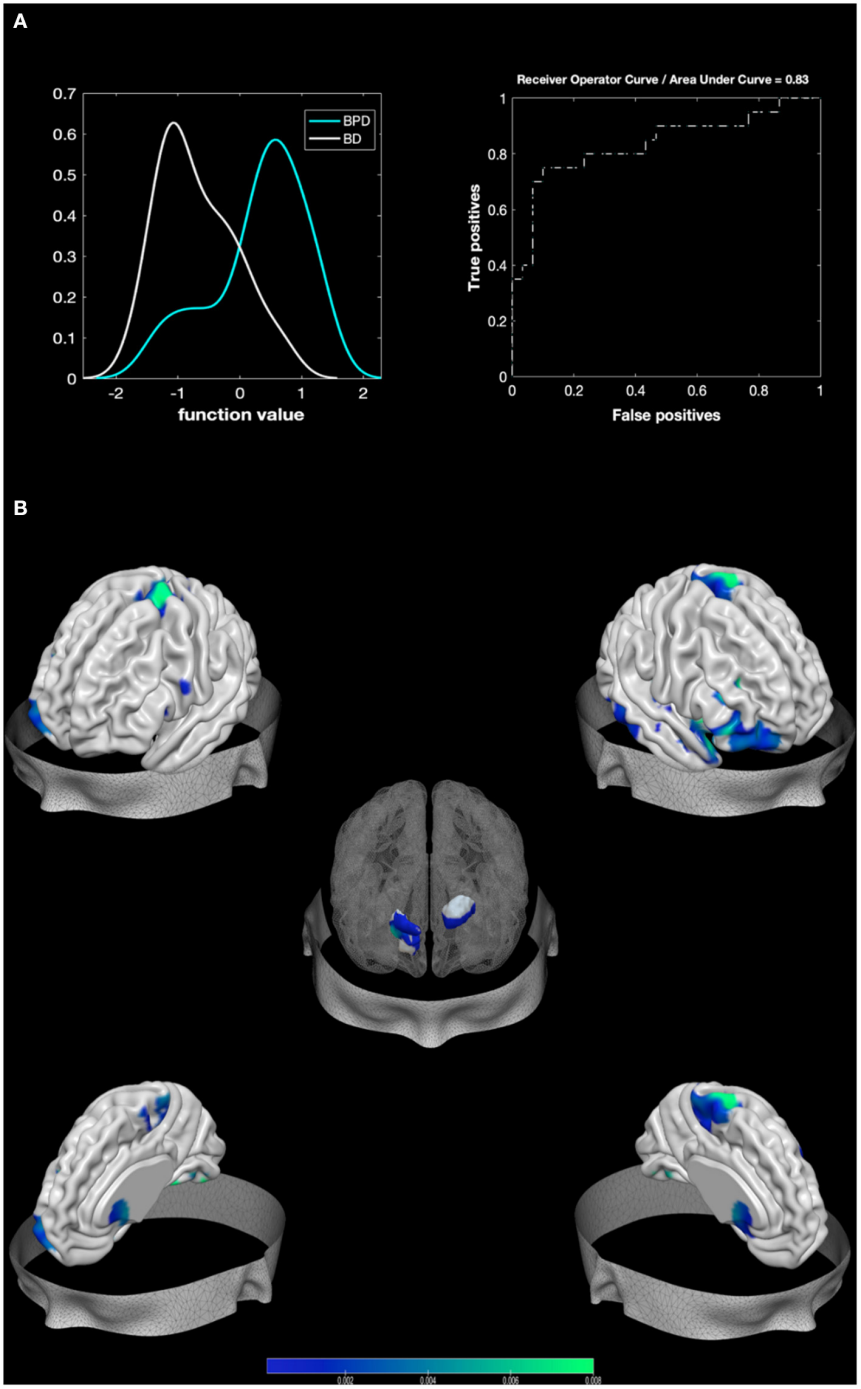
Results from BPD against BD. Multiple Kernel Learning machine classification of patients with Borderline Personality Disorder (BPD) and healthy controls (HC) based on structural (GM) features. **(A)** Left: Density version of histogram plot of function values. Right: Receiver Operator Curve, Areas Under the Curve = 0.83. ROI weights in percentage and in voxel size are displayed in the two bar plots. **(B)** Surface plots, including subcortical reconstruction of the significant regions.

The MKL returned a total accuracy of 80%, BA of 79.17% (*p* = 0.001), class predictive values of 75% for BPD and 83.33% for BD, and AUC value of 83% (Figure 2). Model performance significantly exceeds the threshold of randomly guessing the labels, thus confirming that the algorithm has successfully learned a predictive function (56). Regions with larger contribution to the model (weight > 1%) were in order of importance: the right pallidum, the right inferior frontal cortex, the right amygdala, portions of the cerebellum, the right superior temporal pole, the right fusiform, the right inferior temporal area, the right putamen, the left caudate, and the right superior part of the OFC among others (see Figure 2; Table 3).

**Table 3:** Main regions derived from the classification of BPD against BD.

**ROI label**	**ROI weight %**	**ROI size**
Pallidum_R	19.3420	608
Frontal_Inf_Tri_R	11.4499	3,654
Amygdala_R	9.2810	571
Vermis_6	8.1206	797
Temporal_Pole_sup_R	7.2565	2,085
Fusiform_R	6.5385	5,731
Putamen_R	6.3606	2,560
Tempora_Inf_R	4.2000	7,209
Cerebellum_8_L	4.0807	2,619
Caudate_L	3.8829	2,212
Frontal_Sup_Orb_R	3.7407	1,352
Frontal_Mid_Orb_R	3.0579	1,769
Vermis_4_5	2.9622	1,489
Cerebellum_10_L	2.3693	342
Cerebellum_7b_L	2.2482	863
Frontal_Inf_Oper_L	1.2234	2,479
Thalamus_L	1.0755	2,420

*ROI labels are derived from the AAL atlas. Please note that only regions whose contribution exceeded the 1% are displayed*.

### BPD Against HC and BD

Areas surviving both contrasts (e.g., areas that separate BPD from both control groups) were in order of importance (weight contribution derived from BD against HC model): the right Putamen, the right amygdala, the superior and mid parts of the orbitofrontal, the fusiform area, and the left 7b part of the cerebellum.

## Discussion

The detection of neuroimaging-based biomarkers of BPD may crucially contribute to overcome the limitations of diagnostic procedures exclusively based on subjective evaluations of clinical signs and symptoms and to further elucidate the neural mechanisms of such disorder. With this regard, traditional univariate approaches that identify structural and functional abnormalities in brain regions associated with a mental disorder are not suitable for individual diagnosis, mostly because of large inter-individual variance in regional fMRI activations. To overcome this gap, the first aim of the present study is to apply a multivariate whole-brain machine learning approach to distinguish BPD from HCs on the basis of structural brain features. Yet, to ensure that results are specific for BPD and not for similar mental disorders, the second aim of the present study is to compare the BPD brain alterations with a commonly associated mental disorder that is BD (45, 60).

Overall, results showed that patients with BPD were correctly and reliably classified against HC (total accuracy of 84.62%) and BD (total accuracy of 80%). To our knowledge, this is the first study using machine learning methodology to identify a BPD-specific neural circuit to serve as a possible biomarker based on structural features. The success of the present procedure in predicting BPD against HC and another clinical control group (BD) fosters the machine learning approach as a useful method to allow classification of structural brain images of each patient, in line with other applications of machine learning for clinical diagnosis classifications [for reviews, see the works of Fu and Costafreda (61) and Wolfers et al. (62)].

The most relevant sources for the classification of BPD and HC included structural alterations in several subcortical structures (such as the amygdala, the thalamus, the pallidum, and the putamen), in the fusiform gyrus, in the OFC, and in the cerebellum. Notably, we also observed significant associations between these brain alterations and the affective sector of the Zanarini questionnaire, thus strengthening the association between brain such alterations and affective disturbances characterizing this pathology. The localization of the highest accuracy scores in subcortical regions is consistent with the results of past univariate comparisons of BPD and HC (9, 17, 20, 21). The putamen, which is the structure with the greatest weight in the model, and the pallidum both belong to the basal ganglia and underpin reward processing and impulsivity behaviors (18, 19, 29, 30). In BPD, an increased activity of these brain regions is associated with impulsivity and aggressiveness traits (63), as well as with the processing of negative stimuli and emotion dysregulation (64). Structural alterations of the thalamus have been previously reported in patients with BPD (6, 21, 23). Of note, Nenadić et al. (6) found a negative correlation between the GM concentration of the thalamus and symptoms severity. Our results confirm such reduced GM in the thalamus as well as in the basal ganglia in patients with BPD. The activity in this brain area is linked not only to emotion (65) and reward (66) but also to general mental operations such as attention, memory, and consciousness (67). The amygdala, another region with larger contribution in the model, is probably one of the most targeted areas in structural BPD investigations (9, 24–26). Having a primary role in decoding affective information and in generating emotional arousal and triggering behavioral responses (68, 69), alterations in the amygdala have been extensively reported in BPD (17). Several studies reported an increased responsivity of the amygdala during processing of negative stimuli (9), which suggest a hypersensitivity of this structure in BPD. Such increased responsivity is supported by the fact that, in our additional analyses (raw data voxel-to-voxel comparisons), we also found these areas to have greater GM in BPD as compared with HC.

With regard to cortical areas, the fusiform gyrus was another area highly contributing to the classification. This area is involved in humans' face processing (70) and plays certainly a key role in social cognition (71, 72). Its structural abnormalities in BPD have been already reported in other studies (27), and functional alterations of this area emerged mostly in studies using social stimuli (73), especially emotional faces (27). There is also evidence that fusiform cortex volume correlated positively with insufficient self-control (6). Our further analyses showed increased GM as compared with HC in this area, further supporting the hypothesis of the abnormal activity found in the previous studies.

The OFC, with its anatomical connections with cortical and subcortical limbic areas, is another key area in BPD neuroimaging literature (18, 74). In support of our findings, a recent meta-analysis (45) reported that structural differences between BPD and control in the OFC were robust and consistent across the selected literature (i.e., 13 studies). The OFC has been involved in many humans' behavior among which actions inhibition, and control and regulation of emotional responses (18, 74). Notably, these studies found smaller GM concentration in the medial part of the OFC for BPD compared with controls. Whereas, in our analyses, we found GM increased in the right lateral part of the OFC for BPD compared with control. Future studies are needed to better understand the role of different portions of the OFC in BPD. Other studies reported a negative relationship in the local gyrification of this area and impulsivity in BPD (17, 75). In addition, the OFC and the right inferior frontal gyrus seem to play a key role in anger and its regulation (76), which is known to be dysregulated in BPD.

Finally, in addition, the cerebellum, among other functions, is also involved in affective evaluation, in interaction with the prefrontal cortex and basal ganglia (77). Accordingly, the cerebellar–thalamus–striatum circuit could be linked to reward and mood alterations in BPD (29). In line with previous observations (6), we found increased GM in the cerebellum for BPD compared with HC.

To identify a possible substrate to serve as a future biomarker for BPD, we also compared BPD with another clinical control group of patients with BD. Despite the previous observation of a shared neural substrate between BPD and BD (60), the machine learning approach used in the present study offered the advantage to clearly disentangle the potential ambiguity due to the similarity of clinical signs shared by both disorders, accounting for a neural differentiation between these two pathological conditions (30). With regard to the source of such neural differentiation, again, subcortical brain structures resulted to have the highest discriminative power, suggesting that emotional reactivity (i.e., the amygdala) and impulsivity (i.e., the pallidum, the putamen, and the caudate) that discriminate BPD from HC also discriminate between BPD and BD. The involvement of the striatum in the regulation of voluntary actions as well as in motivated behaviors (78) may represent a coherent substrate for BPD symptomatology related to the impulsivity, intended as aggressiveness, substance abuse, self-mutilation, and eating disorders (60). Abnormalities in the amygdala, on the other hand, explain the hypersensitivity of patients with BPD to emotionally salient stimuli, especially the negative ones [e.g., (9)], and the typical rapid-cycling mood changes in these patients.

The cluster of abnormalities in brain regions involved in socio-affective processes may be a further aspect distinguishing BPD from BD. Indeed, a specific deficit in BPD, which is only marginal in BD, concerns the handling of social relationships (1). In BPD, interpersonal experiences are often characterized “by rapid attachment, anxious dependency, and fear of abandonment” [(1), p. 4]. According to this social deficiency, the alteration characterizing BPD embraced the temporal regions and the fusiform area, both associated to the processing of socio-affective stimuli (emotional faces). Similarly, the vermis is considered as the limbic portion of the cerebellum, which, in turn, has been recently suggested a structure related to social brain (79).

**Figure 3:**
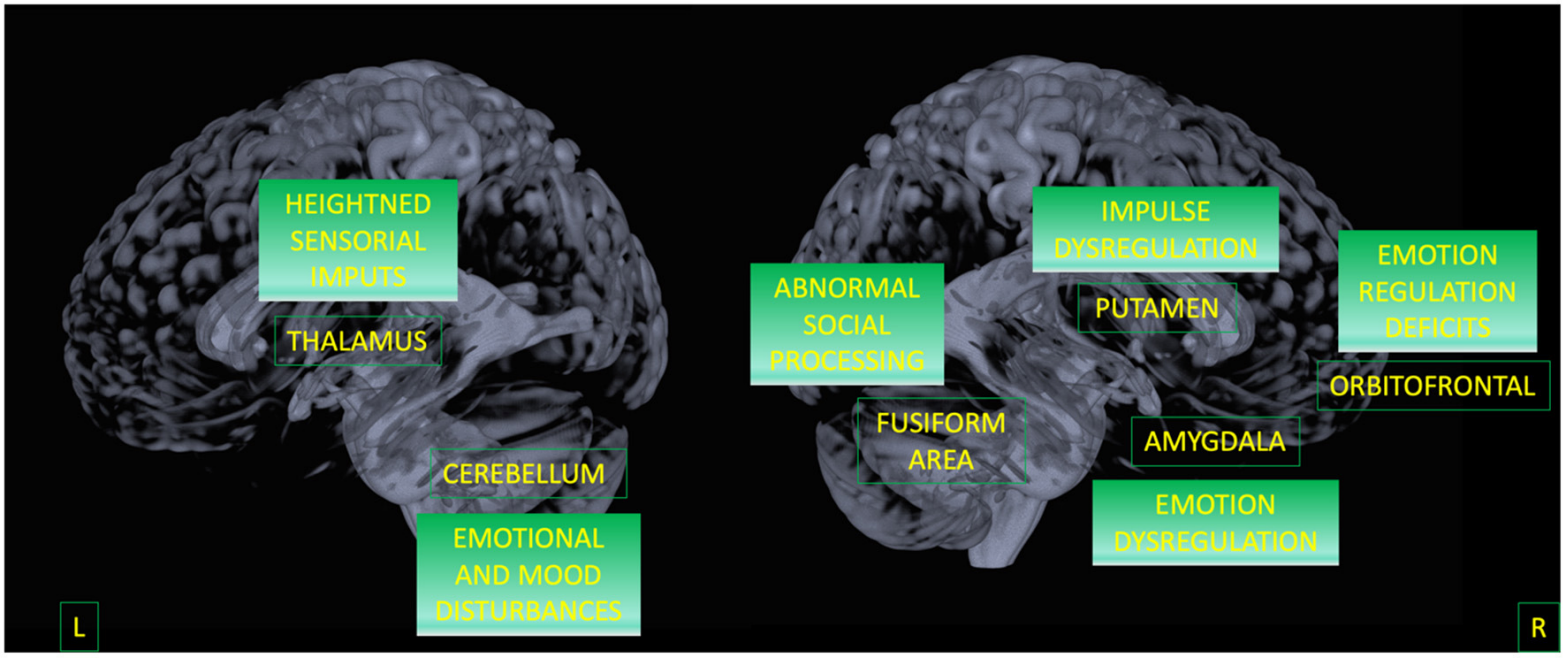
A model for BPD circuit. Areas surviving both contrasts (BPD against HC, and BPD against BD) are displayed as well as their potential functional meaning.

Finally, by considering only the regions that discriminate BPD from both HC and BD, we were able to outline a possible biomarker specific for BPD. Results indicate that the alterations in the triad involving putamen, amygdala, and OFC represent the specificity of this disorder (42–44). This circuit has been largely associated with affective instability and anger control as a consequence of dysfunctional emotion regulation (see Figure 3) (3, 10, 11, 29, 30, 80, 81).

The present study provides the first evidence of a specific neural circuit that correctly classify patients with BPD, but such a scientific advance does not come without some limitations to point out. First, the sample size of patients with BPD was small. This is mainly because it is difficult to find open datasets including BPD data. For other disorders such as schizophrenia, depression, and anxiety, large datasets are now publicly available, but not for personality disorders. We acknowledged that a limited sample size in predictive models has been recently questioned because it may affect cross-validation error (82). However, there is also evidence that bigger sample size does not improve bias in performance estimates when this validation method is used [(83); see also (84), for different considerations on this topic]. Future studies with larger sample are needed to replicate these findings. A second limitation concerns mostly the type of the sample that consisted in patients on drugs. Although this can be a factor to be controlled in functional studies, in structural investigations, it should less affect results and individual variability (28, 31).

Despite the limitations, the present study represents a first attempt to addressed scholars' concerns that mental disorders are still lacking reliable and specific biomarkers (85–87), especially when considering BPD and personality disorders (88–90). The present finding encourages the employment of this methodology to disclose pathophysiology and improve classification of mental diseases (85, 89). We hope that this study will inspire future investigations in which a larger sample size will be used and in which BPD will be also distinguished from other psychopathologies, such as schizophrenia (91) or major depression (92).

## Data Availability Statement

Publicly available datasets were analyzed in this study. This data can be found at: UCLA Consortium for Neuropsychiatric Phenomics - OpenNeuro database, accession number ds000030.

## Ethics Statement

The studies involving human participants were reviewed and approved by reuse of open access data, see details inside manuscript. The patients/participants provided their written informed consent to participate in this study.

## Author Contributions

AG conceptualized the study, performed the machine learning and other statistical analyses, and drafted the manuscript. GL and SS pre-processed the data and revised the manuscript. BM and IM conceptualized the study, drafted, and critically reviewed the article. RS supervised and reviewed the article. All authors contributed to the article and approved the submitted version.

## Conflict of Interest

The authors declare that the research was conducted in the absence of any commercial or financial relationships that could be construed as a potential conflict of interest.

## Publisher's Note

All claims expressed in this article are solely those of the authors and do not necessarily represent those of their affiliated organizations, or those of the publisher, the editors and the reviewers. Any product that may be evaluated in this article, or claim that may be made by its manufacturer, is not guaranteed or endorsed by the publisher.
